# Idiopathic Chronic Eosinophilic Pneumonia

**DOI:** 10.7759/cureus.14047

**Published:** 2021-03-22

**Authors:** Natália Teixeira, Maria Inês Santos, Filipa Pedro, Maria João Pinto, Ana Mestre

**Affiliations:** 1 Internal Medicine, Hospital Distrital de Santarém, Santarém, PRT

**Keywords:** corticosteroid therapy, dyspnea, eosinophilic pneumonia, idiopathic chronic eosinophilic pneumonia, pneumonia

## Abstract

Idiopathic chronic eosinophilic pneumonia (CEP) is a rare disease of unknown cause characterized by eosinophilic alveolar and interstitial infiltration. The authors describe the case of a 46-year-old black man, presenting with insidious onset and progressive course of dyspnea on minimum exertion, cough, fever, night sweats, and weight loss for one year and worsening in the last three months. The main findings were serum eosinophilia. Chest radiographs showed multifocal infiltrations of irregular distribution in both lungs and a restrictive functional impairment. The patient underwent open lung biopsy, and the anatomopathological examination revealed consolidation by exudate constituted predominantly by macrophages (25%) and eosinophils (51%), which filled small air spaces, including respiratory and membranous bronchioles. The anatomopathological diagnosis was eosinophilic pneumonia (eosinophils > 25% is widely accepted for diagnosing eosinophilic pneumonia). The patient had a good clinical response after starting corticosteroid therapy.

## Introduction

Eosinophilic pneumonias (EP) represent a heterogeneous group of pulmonary diseases characterized by infiltration of eosinophils into the lung compartments (airways, interstitium, and alveoli), with or without evidence of eosinophilia in the peripheral blood [1,2]. Idiopathic chronic eosinophilic pneumonia (CEP) is a rare clinical entity characterized by alveolar and interstitial infiltration of eosinophils of unknown etiology. It has a peak incidence in females aged between 30 and 50 years old. CEP has an insidious onset, a chronic and prolonged course, and presents with nonspecific, constitutional complaints, such as fever, night sweats, and weight loss. The objective examination is usually nonspecific, and the laboratory findings are eosinophilia (90%), leukocytosis (60%-90%), and increased erythrocyte sedimentation rate (ESR) (50%-70%). From a functional point of view, it is defined by a moderate to severe restrictive spirometry pattern [3,4]. Imaging is characterized by the presence of dense, peripheral, ill-defined, non-migrating alveolar opacities. The distribution is usually bilateral and symmetric [1]. CEP lesions are histologically characterized by alveolar infiltrates in the air space and interstitium, with a predominance of eosinophils. The normal alveolar wall architecture is altered, with focal edema and endothelial hyperplasia. The diagnosis of CEP is based on clinical and laboratory findings and response to corticosteroid therapy. Although the prognosis of treated patients with CEP is excellent, most will require continued therapy with low doses of oral corticosteroids to prevent recurrence. The authors describe the case of a man with a history of asthma, presenting with dyspnea, fever, and constitutional symptoms, who arrived at the diagnosis of CEP. We will discuss in this article the etiology, differential diagnosis, and particularities of this situation.

## Case presentation

**Figure 1:**
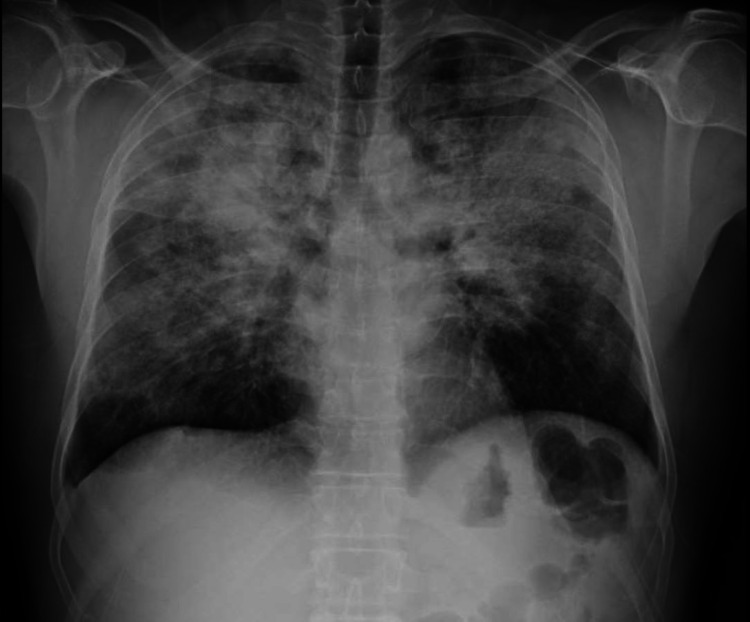
Chest x-ray Chest x-ray showing several foci of consolidation scattered throughout the lung parenchyma.

A 46-year-old male from Cape Verde with a history of asthma and a history of occupational exposure to paints and solvents presented to emergency department with complaint of dyspnea insidious onset and progressive course of night sweats, febrile, dry cough, and unquantified weight loss in the last year. He currently uses an inhaled steroid-long acting bronchodilator (ICS-LABA) daily. On the objective exam he was found to be tachypneic, with oxygen saturation of 94% on room air, sweating, and febrile. On pulmonary auscultation, diminished breath sounds were present, and there were scattered crackling sounds. Lab results showed the following: hemoglobin 11.6 g/dL; elevation of inflammatory parameters with C-reactive protein 21 mg/dL; and leukocytosis with predominance of eosinophilia (37.4%). Viral serology and baciloscopy on direct sputum examination did not isolate any organism. Arterial blood gas (FiO2 21%) revealed hypoxemia with pO2 62 mmHg. Lung radiography shown in Figure 1 revealed bilateral opacities at the periphery of the lung fields.

**Figure 2:**
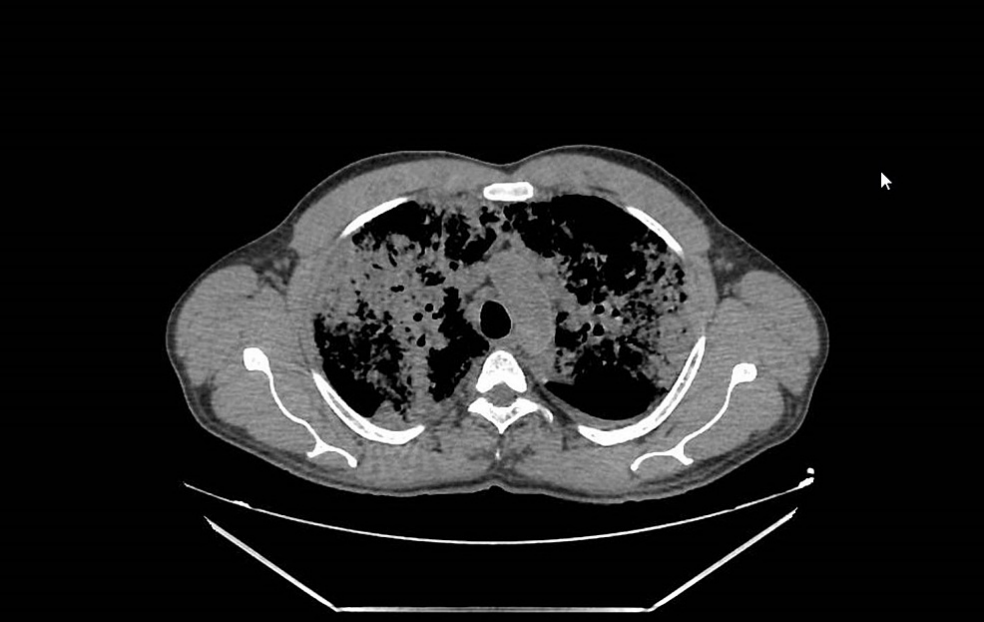
Chest CT Chest CT showing thickened bronchial walls, areas of ground glass in the upper lobes, and peripheral predominance, assuming the appearance of consolidation in some areas. CT, Computed tomography.

The computed tomography (CT) scan of the chest revealed extensive ground-glass opacification of the lung parenchyma and consolidation with air bronchogram, bilateral and plurilobar, without cavitations (Figure 2).

**Figure 3:**
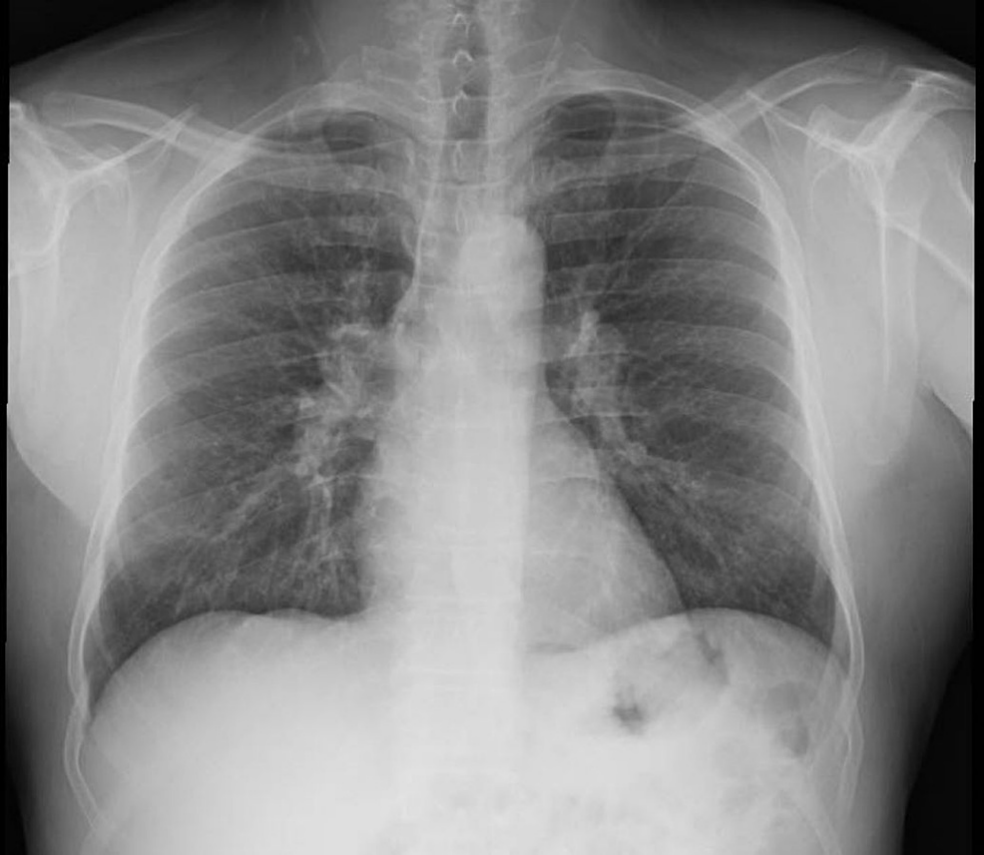
Chest x-ray (reevaluation) Reevaluation chest x-ray (three months later) showing clear radiological improvement.

The CT also showed evidence of irregular bronchial dilatations associated with established fibrosis. Bronchofibroscopy showed no endobronchial changes. Bronchoalveolar lavage fluid (BAL) showed an eosinophilic alveolitis (total cell count of 139/mm^3^, with 51% eosinophils and a CD4/CD8 ratio of 1:1). The bronchial aspirate did not isolate any organism, and its cytology was composed of reactive bronchial ciliated cells and inflammatory cells. There were no neoplastic cells. Parasitological examination of stool was negative. The IgE and angiotensin-converting enzyme levels were within range, and the immunological study had no significant abnormalities. Histology of the transbronchial biopsy specimen (left upper lobe) revealed a flap of loose connective tissue with scattered epithelial cells, not allowing its diagnostic recognition by this examination. Once the diagnosis of CEP was established, the patient was medicated with prednisolone 1 mg/kg/day. There was not only a complete symptomatic resolution but also a clinical, radiological, and functional response after the initial three months of treatment (Figure 3).

## Discussion

EP represent a heterogeneous group of lung diseases characterized by alveolar eosinophilia and pulmonary infiltrates, with or without evidence of eosinophilia in the peripheral blood [1,2]. They are classified into primary (idiopathic) and secondary, according to the identification of the specific etiological context and the clinical imaging patterns [1]. CEP is a rare clinical entity characterized by alveolar and interstitial infiltration of eosinophils (BAL with eosinophils > 25% is widely accepted for diagnosing eosinophilic pneumonia) of unknown etiology. Autoimmune or hypersensitivity reaction has been strongly implicated in its origin. About two-thirds of patients have a history of atopy, and about 50% of cases report a previous history of asthma [5-7]. CEP affects twice as many women as men and is usually diagnosed in the fifth decade of life [1]. Symptoms may present several months before diagnosis and manifest as progressive dyspnea, dry cough, and constitutional symptoms (fever, asthenia, night sweats, and weight loss). Cough, which is usually dry, is the most frequent symptom followed by dyspnea, which is usually mild or moderate. Additional non-respiratory symptoms in individuals with CEP are uncommon. However, joint pain, nerve damage, and general skin or gastrointestinal symptoms have been reported in the medical literature. The objective examination is usually nonspecific, showing wheezing or crackles on pulmonary auscultation in about one-third of the patients. Sputum and BAL eosinophilia are frequent, but peripheral eosinophilia and elevated serum IgE are not present in all cases [6]. Peripheral eosinophilia and elevated serum IgE are present in 60%-90% of cases [7,8]. Serum IgE levels are elevated in 50%-60% of patients (up to 1000 IU/ml), reflecting the large percentage of cases with an atopic background, and increased inflammatory parameters may also be observed.

Most patients with CEP present a characteristic radiological picture, which is virtually pathognomonic and consists of homogeneous opacities, with irregular margins, not respecting the anatomical barriers, therefore with a radiographic pattern suggestive of airspace consolidation. The opacities, usually lateral, are arranged, as already mentioned, in the periphery and may involve the entire lung, giving rise to an image that has been described as a photographic negative of pulmonary edema or also as the inverted pattern of bat wings. The upper and middle lung fields are preferentially affected [5,6]. Other pulmonary findings such as adenopathy, cavitation, atelectasis, and pleural effusion are infrequent [8]. In the functional study, the restrictive pattern is observed in more than 75% of patients, and there is disturbance of gas diffusion through the alveolocapillary membrane, evidenced by hypoxemia in about two-thirds of patients, and hypocapnia may be associated [7]. 

The diagnosis of CEP is based on a detailed anamnesis complemented by laboratory tests, in which the BAL is fundamental, imaging tests, and the exclusion of other pathologies associated with pulmonary eosinophilia [9,10]. In a small percentage of cases (<5%), a lung biopsy may be necessary, and lung histology reveals interstitial and alveolar eosinophils and histiocytes, including multinucleated giant cells. The differential diagnosis of CEP includes infections (by mycobacteria and fungal diseases such as cryptococcosis), sarcoidosis, Loeffler syndrome, Langerhans cell histiocytosis, desquamative interstitial pneumonia, bronchiolitis obliterans with organizing pneumonia, chronic hypersensitivity pneumonitis, and Wegener's granulomatosis [5]. In Loeffler syndrome, and unlike CEP, pulmonary infiltrates are migratory. Corticotherapy is the mainstay of treatment, and a rapid response to corticosteroids is characteristic in CEP (after one week of treatment the resorption of lesions is considerable, sometimes obtaining a normal radiological picture after one month). In many cases, response to the therapeutic is used to establish the diagnosis. Currently, initial treatment is with prednisolone at a dose of approximately 40-60 mg/day for 10-14 days, followed by a gradual de-escalation regimen [1,11]. With discontinuation of corticotherapy, recurrent relapses of the disease may occur; however, disease relapses remain with good response to corticotherapy [6,7,12]. CEP presents a generally favorable clinical course [13,14]. Progression to pulmonary fibrosis has been described, but such evolution seems to be rare [15]. In the present case, the diagnostic hypothesis of CEP was established based on the compatible clinical history, peripheral eosinophilia and BAL, suggestive pulmonary infiltrate on imaging study, absence of evident infection, and response to corticosteroid use.

## Conclusions

CEP is a rare interstitial lung disease associated with eosinophilic alveolar infiltration with significant morbidity, but rapid diagnosis and treatment with corticosteroids should be considered in the proper clinical scenario. In the present case, clinical, laboratory (especially BAL), and radiographic data allowed the diagnosis of CEP. The authors intend this study appeal to the rarity of the disease and the necessity of a high index of suspicion in order to avoid underdiagnosis as a correct early diagnosis and adequate therapy are fundamental elements for a better prognosis of these patients.
